# An integrated pharmacokinetics ontology and corpus for text mining

**DOI:** 10.1186/1471-2105-14-35

**Published:** 2013-02-01

**Authors:** Heng-Yi Wu, Shreyas Karnik, Abhinita Subhadarshini, Zhiping Wang, Santosh Philips, Xu Han, Chienwei Chiang, Lei Liu, Malaz Boustani, Luis M Rocha, Sara K Quinney, David Flockhart, Lang Li

**Affiliations:** 1 Center for Computational Biology and Bioinformatics, School of Medicine, Indiana University, Indianapolis, IN, USA; 2 Department of Medical and Molecular Genetics, School of Medicine, Indiana University, Indianapolis, IN, USA; 3 Department of Pharmacology and Toxicology, School of Medicine, Indiana University, Indianapolis, IN, USA; 4 Division of Clinical Pharmacology, School of Medicine, Indiana University, Indianapolis, IN, USA; 5 Shanghai Center for Bioinformation and Technology, Shanghai, 200235, China; 6 Regenstrief Institute, Indianapolis, IN, USA; 7 Informatics and Cognitive Science Center for Complex Networks and Systems Research, School of Informatics & Computing, Indianapolis, IN, USA; 8 Indiana Institute of Personalized Medicine, Indianapolis, IN, USA; 9 Department of Obstetrics and Gynecology, School of Medicine, Indiana University, Indianapolis, IN, USA

## Abstract

**Background:**

Drug pharmacokinetics parameters, drug interaction parameters, and pharmacogenetics data have been unevenly collected in different databases and published extensively in the literature. Without appropriate pharmacokinetics ontology and a well annotated pharmacokinetics corpus, it will be difficult to develop text mining tools for pharmacokinetics data collection from the literature and pharmacokinetics data integration from multiple databases.

**Description:**

A comprehensive pharmacokinetics ontology was constructed. It can annotate all aspects of *in vitro* pharmacokinetics experiments and *in vivo* pharmacokinetics studies. It covers all drug metabolism and transportation enzymes. Using our pharmacokinetics ontology, a PK-corpus was constructed to present four classes of pharmacokinetics abstracts: *in vivo* pharmacokinetics studies, *in vivo* pharmacogenetic studies, *in vivo* drug interaction studies, and *in vitro* drug interaction studies. A novel hierarchical three level annotation scheme was proposed and implemented to tag key terms, drug interaction sentences, and drug interaction pairs. The utility of the pharmacokinetics ontology was demonstrated by annotating three pharmacokinetics studies; and the utility of the PK-corpus was demonstrated by a drug interaction extraction text mining analysis.

**Conclusions:**

The pharmacokinetics ontology annotates both *in vitro* pharmacokinetics experiments and *in vivo* pharmacokinetics studies. The PK-corpus is a highly valuable resource for the text mining of pharmacokinetics parameters and drug interactions.

## Background

Pharmacokinetics (PK) is a very important translational research field, which studies drug absorption, disposition, metabolism, excretion, and transportation (ADMET). PK systematically investigates the physiological and biochemical mechanisms of drug exposure in multiple tissue types, cells, animals, and human subjects [1]. There are two major molecular mechanisms of a drug’s PK: metabolism and transportation. The drug metabolism mainly happens in the gut and liver; while drug transportation exists in all tissue types. If the PK can be interpreted as how a body does on the drug, pharmacodynamics (PD) can be defined as how a drug does on the body. A drug’s pharmacodynamics effect ranges widely from the molecular signals (such as its targets or downstream biomarkers) to clinical symptoms (such as the efficacy or side effect endpoints) [1].

Drug-drug interaction (DDI) is another important pharmacology concept. It is defined as whether one drug’s PK or PD response is changed due to the presence of another drug. PD based drug interaction has a wide range of interpretations (i.e. from molecular markers to clinical endpoints). PK based drug interaction mechanism is very well defined: metabolism enzyme based and transporter based DDIs. Pharmacogenetic (PG) variations in a drug’s PK and PD pathways can also affect its responses [1]. In this paper, we will focus our discussion on the PK, PK based DDI, and PK related PG.

Although significant efforts have been invested to integrate biochemistry, genetics, and clinical information for drugs, significant gaps exist in the area of PK. For example DrugBank (http://www.drugbank.ca/) doesn’t have *in vitro* PK and its associated DDI data; DiDB (http://www.druginteractioninfo.org/) doesn’t have sufficient PG data; and PharmGKB (http://www.pharmgkb.org/) doesn’t have sufficient *in vivo* and *in vitro* PK and its associated DDI data. As an alternative approach to collect PK from the published literature, text mining has just started to be explored [1-4].

From either database construction or literature mining, the main challenge of PK data integration is the lack of PK ontology. This paper developed a PK ontology first. Then, a PK corpus was constructed. It facilitated DDI text mining from the literature.

## Construction and content

PK Ontology is composed of several components: experiments, metabolism, transporter, drug, and subject (Table 1). Our primary contribution is the ontology development for the PK experiment, and integration of the PK experiment ontology with other PK-related ontologies.

**Table 1:** PK ontology categories

**Categories**	**Description**	**Resources**
Pharmacokinetics Experiments	Pharmacokinetics studies and parameters. There are two major categories: *in vitro* experiments and *in vivo* studies.	Manually accumulated from text books and literatures.
Transporters	Drug transportation enzymes	http://www.tcdb.org
Metabolism Enzymes	Drug metabolism enzymes	http://www.cypalleles.ki.se/
Drugs	Drug names	http://www.drugbank.ca/
Subjects	Subject description for a pharmacokinetics study. It is composed three categories: disease, physiology, and demographics	http://bioportal.bioontology.org/ontologies/42056
		http://bioportal.bioontology.org/ontologies/39343
		http://bioportal.bioontology.org/ontologies/42067

*Experiment* specifies *in vitro* and *in vivo* PK studies and their associated PK parameters. Table 2 presents definitions and units of the *in vitro* PK parameters. The PK parameters of the single drug metabolism experiment include Michaelis-Menten constant (K_m_), maximum velocity of the enzyme activity (V_max_), intrinsic clearance (CL_int_), metabolic ratio, and fraction of metabolism by an enzyme (fm_enzyme_) [5]. In the transporter experiment, the PK parameters include apparent permeability (Papp), ratio of the basolateral to apical permeability and apical to basolateral permeability (Re), radioactivity, and uptake volume [6]. There are multiple drug interaction mechanisms: competitive inhibition, non-competitive inhibition, uncompetitive inhibition, mechanism based inhibition, and induction [7]. IC_50_ is the inhibition concentration that inhibits to 50% enzyme activity; it is substrate dependent; and it doesn’t imply the inhibition mechanism. K_i_ is the inhibition rate constant for competitive inhibition, noncompetitive inhibition, and uncompetitive inhibition. It represents the inhibition concentration that inhibits to 50% enzyme activity, and it is substrate concentration independent. K_deg_ is the degradation rate constant for the enzyme. K_I_ is the concentration of inhibitor associated with half maximal Inactivation in the mechanism based inhibition; and K_inact_ is the maximum degradation rate constant in the presence of a high concentration of inhibitor in the mechanism based inhibition. E_max_ is the maximum induction rate, and EC_50_ is the concentration of inducer that is associated with the half maximal induction

**Table 2:** ***In vitro *PK parameters**

**Experiment types**	**Parameters**	**Description**	**Unit**	**References**
Single Drug Metabolism Experiment	K_m_	Michaelis-Menten constant.	mg L^-1^	Segel p28.
	V_max_	Maximum velocity of the enzyme activity.	mg h^-1^ mg^-1^ protein	Segel p19
	CL_int_	Intrinsic metabolic clearance is defined as ratio of maximum metabolism rate, Vmax, and the Michaelis-Menten constant, Km.	ml h^-1^ mg^-1^ protein	RT p165
	Metabolic ratio	Parent drug/metabolite concentration ratio	NA	
	fm_enzyme_	Fraction of drug systemically available that is converted to a metabolite through a specific enzyme.	NA	RT xiii
Single Drug Transporter Experiment	Papp	The apparent permeability of compounds across the monolayer cells.	cm/sec	Transport Consortium
	Re	Re is the ratio of basolateral to apical over apical to basolateral.	NA	Transport Consortium
	Radioactivity	Total radioactivity in plasma and bile samples is measured in a liquid scintillation counter	dpm/mg protein	Transport Consortium
	Uptake Volume	The amount of radioactivity associated with the cells divided by its concentration in the incubation medium.	ul/mg protein	Transport Consortium
Drug Interaction Experiment	IC_50_	Inhibitor concentration that inhibits to 50% of enzyme activity.	mg L^-1^	
	K_i_	Inhibition rate constant for competitive inhibition, noncompetitive inhibition, and uncompetitive inhibition.	mg L^-1^	Segel p103
	K_deg_	The natural degradation rate constant for the Enzyme.	h^-1^	Rostami-Hodjegan and Tucker
	K_I_	The concentration of inhibitor associated with half maximal Inactivation in the mechanism based inhibition.	mg L^-1^	Rostami-Hodjegan and Tucker
	K_inact_	The maximum degradation rate constant in the presence of a high concentration of inhibitor in the mechanism based inhibition.	h^-1^	Rostami-Hodjegan and Tucker
	E_max_	Maximum induction rate	Unit free	Rostami-Hodjegan and Tucker
	EC_50_	The concentration of inducer that is associated with the half maximal induction.	mg L^-1^	Rostami-Hodjegan and Tucker
Type of Drug Interactions	Competitive inhibition, noncompetitive inhibition, uncompetitive inhibition, mechanism based inhibition, and induction.	Rostami-Hodjegan and Tucker		

Note: Segel H. Irwin. Enzyme Kinetics – Behavior and analysis of rapid equilibrium and steady state enzyme systems. John Wiley & Sons, Inc. 1975, New York. Rostami-Hodjegan Amin and Tucker Geoff ‘In silico’ simulations to assess the ‘in vivo’ consequences of ‘in vitro’ metabolic drug-drug interactions. Drug Discovery Today, 2004, 1, 441–448. The International Transporter Consortium, Membrane transporters in drug development. Nature Review Drug Discovery, 9, 215–236. Rowland Malcolm and Tozer N. Thomas Clinical Pharmacokinetics Concepts and Applications, 3^rd^ edition. 1995, Lippincott Williams & Wilkins.

The *in vitro* experiment conditions are presented in Table 3. Metabolism enzyme experiment conditions include buffer, NADPH sources, and protein sources. In particular, protein sources include recombinant enzymes, microsomes, hepatocytes, and etc. Sometimes, genotype information is available for the microsome or hepatocyte samples. Transporter experiment conditions include bi-directional transporter, uptake/efflux, and ATPase. Other factors of *in vitro* experiments include pre-incubation time, incubation time, quantification methods, sample size, and data analysis methods. All these info can be found in the FDA website (http://www.abclabs.com/Portals/0/FDAGuidance_DraftDrugInteractionStudies2006.pdf).

**Table 3:** ***In vitro *experiment conditions**

***Experimental Conditions:***	**Drugs**	**Substrate, metabolite, and inhibitor/inducer**			**FDA Drug Interaction Guidance, 2006**
Metabolism Enzymes	Buffer	Salt composition			
		EDTA concentration			
		MgCl_2_ concentration Cytochrome b_5_ concentration			
	NADPH source	Concentration of exogenous NADPH added isocytrate dehydrogenase + NADP			
	protein	Non-recombinant enzymes	Microsomes (human liver microsomes, human intestine microsomes, S9 fraction, cytosol, whole cell lysate, hepatocytes)		
		Recombinant enzymes	Enzyme name	mg/mL or uM	
			genotype		
Transporters	Bi-Directional	CHO; Caco-2 cells; HEK-293; Hepa-RG; LLC; LLC-PK1 MDR1 cells; MDCK; MDCK-MDR1 cells; Suspension Hepatocyte			
	Transport				
	Uptake/efflux	tumor cells, cDNA transfected cells, oocytes injected with cRNA of transporters			
	ATPase	membrane vesicles from various tissues or cells expressing P-gp, Reconstituted P-gp			
Other factors	Pre-incubation time				
	Incubation time				
	Quantification methods	HPLC/UV, LC/MS/MS, LC/MS, radiographic			
	Sample size				
	Data Analysis	log-linear regression, plotting; and nonlinear regression			

The *in vivo* PK parameters are presented in Table 4. All of the information are summarized from two text books [1,8]. There are several main classes of PK parameters. Area under the concentration curve parameters are (AUC_inf_, AUC_SS_, AUC_t_, AUMC); drug clearance parameters are (CL, CL_b_, CL_u_, CL_H_, CL_R_, CL_po_, CL_IV_, CL_int_, CL_12_); drug concentration parameters are (C_max_, C_SS_); extraction ratio and bioavailability parameters are (E, E_H_, F, F_G_, F_H_, F_R_, f_e_, f_m_); rate constants include elimination rate constant k, absorption rate constant ka, urinary excretion rate constant ke, Michaelis-Menten constant Km, distribution rate constants (k_12_, k_21_), and two rate constants in the two-compartment model (λ_1_, λ_2_); blood flow rate (Q, Q_H_); time parameters (t_max_, t_1/2_); volume distribution parameters (V, V_b_, V_1_, V_2_, V_ss_); maximum rate of metabolism, Vmax; and ratios of PK parameters that present the extend of the drug interaction, (AUCR, CL ratio, Cmax ratio, C_ss_ ratio, t_1/2_ ratio).

**Table 4:** ***In vivo *PK studies**

**Category**	**Name**	**Description**	**Unit**	**reference**
PK parameters	AUC_inf_	Area under the drug concentration time curve.	mg h L^-1^	RT p37
	AUC_SS_	Area under the drug concentration time curve within a dosing curve at steady state.	mg h L^-1^	RT pxi
	AUC_t_	Area under the drug concentration time curve from time 0 to t.	mg h L^-1^	RT p37
	AUMC	Area under the first moment of concentration versus time curve.	mg^2^ h L^-2^	RT p486
	AUCR	AUC ratio (drug interaction parameter).	Unit free	
	CL	Total clearance is defined as the proportionality factor relating rate of drug elimination to the plasma drug concentration.	ml h^-1^	RT p23
	CL_b_	Blood clearance is defined as the proportionality factor relating rate of drug elimination to the blood drug concentration.	ml h^-1^	RT p160
	CL_u_	Unbound clearance is defined as the proportionality factor relating rate of drug elimination to the unbounded plasma drug concentration.	ml h^-1^	RT p163
	CL_H_	Hepatic portion of the total clearance.	ml h^-1^	RT p161
	CL_R_	Renal portion of the total clearance.	ml h^-1^	RT p161
	CL_po_	Total clearance of drug following an oral dose.	ml h^-1^	
	CL_IV_	Total clearance of drug following an IV dose.	ml h^-1^	
	CL_int_	Intrinsic metabolic clearance is defined as ratio of maximum metabolism rate, Vmax, and the Michaelis-Menten constant, Km.	ml h^-1^	RT p165
	CL_12_	Inter-compartment distribution between the central compartment and the peripheral compartment.	ml h^-1^	
	CL ratio	Ratio of the clearance (drug interaction parameter).	Unit free	
	C_max_	Highest drug concentration observed in plasma following administration of an extravascular dose.	mg L^-1^	RT pxii
	C_max_ ratio	The ratio of C_max_ (drug interaction parameter).	Unit free	
	C_ss_	Concentration of drug in plasma at steady state during a constant rate intravenous infusion.	mg L^-1^	RT pxii
	C_ss_ ratio	The ratio of C_ss_ (drug interaction parameter).	Unit free	
	E	Extraction ratio is defined as the ratio between blood clearance, CL_b_, and the blood flow.	Unit free	RT p159
	E_H_	Hepatic extraction ratio.	Unit free	RT p161
	F	Bioavailability is defined as the proportion of the drug reaches the systemic blood.	Unit free	RT p42
	F_G_	Gut-wall bioavailability.	Unit free	
	F_H_	Hepatic bioavailability.	Unit free	RT p167
	F_R_	Renal bioavailability.	Unit free	RT p170
	fe	Fraction of drug systemically available that is excreted unchanged in urine.	Unit free	RT pxiii
	fm	Fraction of drug systemically available that is converted to a metabolite.	Unit free	RT pxiii
	fu	Ratio of unbound and total drug concentrations in plasma.	Unit free	RT pxiii
	k	Elimination rate constant.	h^-1^	RT pxiii
	K_12_, k_21_	Distribution rate constants between central compartment and peripheral compartment.	h^-1^	
	ka	Absorption rate constant.	h^-1^	RT pxiii
	ke	Urinary excretion rate constant.	h^-1^	RT pxiii
	km	Rate constant for the elimination of a metabolite.	h^-1^	RT pxiii
	Km	Michaelis-Menten constant.	mg L^-1^	RT pxiii
	MRT	Mean time a molecular resides in body.	h	RT pxiv
	Q	Blood flow.	L h^-1^	RT pxiv
	Q_H_	Hepatic blood flow.	L h^-1^	RT pxiv
	t_max_	Time at which the highest drug concentration occurs following administration of an extravascular dose.	h	RT pxiv
	t_1/2_	Half-life of the drug disposition.	h	RT pxiv
	t_1/2_ ratio	Half-life ratio (drug interaction parameter).	Unit free	
	t_1/2,α_	Half-life of the fast phase drug disposition.	h	
	t_1/2,β_	Half-life of the slow phase drug disposition.	h	
	V	Volume of distribution based on drug concentration in plasma.	L	RT pxiv
	V_b_	Volume of distribution based on drug concentration in blood.	L	RT pxiv
	V_1_	Volume of distribution of the central compartment.	L	RT pxiv
	V_2_	Volume of distribution of the peripheral compartment.	L	
	V_ss_	Volume of distribution under the steady state concentration.	L	RT pxiv
	Vmax	Maximum rate of metabolism by an enzymatically mediated reaction.	mg h^-1^	RT pxiv
	λ_1_, λ_2_	Disposition rate constants in a two-compartment model.	h^-1^	GP p84
Pharmacokinetics Models	Non-Compartment	Use drug concentration measurements directly to estimate PK parameters, such as AUC, CL, C_max_, T_max_, t_1/2_, F, and V.		GP p409
	One Compartment Model	It assumes the whole body is a homogeneous compartment, and the distribution of the drug from the blood to tissue is very fast. It assumes either a first order or a zero order absorption rate and a first order eliminate rate. Its PK parameters include (ka, V, CL, F).		RT p34
				GP p1
	Two Compartment Model	It assumes the whole body can be divided into two compartments: central compartment (i.e. systemic compartment) and peripheral compartment (i.e. tissue compartment). It assumes either a first order or a zero order absorption rate and a first order eliminate and distribution rates. Its PK parameters include (ka, V_1_, V_2_, CL, CL_12_, F).		GP p84
Study Designs	Hypothesis	Bioequivalence, drug interaction, pharmacogenetics, and disease conditions.		
	Design	Single arm or multiple arms; cross-over or fixed order design; with or without randomization; with or without stratification; prescreening or no-prescreening; prospective or retrospective studies; and case reports or cohort studies.		
	Sample size	The number of subjects, and the number of plasma or urine samples per subject.		
	Time points	Sampling time points and dosing time points.		
	Sample types	Blood, plasma, and urine.		
	Dose	Subject specific doses.		
Quantification methods	HPLC/UV, LC/MS/MS, LC/MS, radiographic			

Rowland Malcolm and Tozer N. Thomas Clinical Pharmacokinetics Concepts and Applications, 3^rd^ edition. 1995, Lippincott Williams & Wilkins.

Gibaldi Milo and Perrier Donald. Pharmacokinetics, 2^nd^ edition. 1982, Dekker.

It is also shown in Table 4 that two types of pharmacokinetics models are usually presented in the literature: non-compartment model and one or two-compartment models. There are multiple items need to be considered in an *in vivo* PK study. The hypotheses include the effect of bioequivalence, drug interaction, pharmacogenetics, and disease conditions on a drug’s PK. The design strategies are very diverse: single arm or multiple arms, cross-over or fixed order design, with or without randomization, with or without stratification, pre-screening or no-pre-screening based on genetic information, prospective or retrospective studies, and case reports or cohort studies. The sample size includes the number of subjects, and the number of plasma or urine samples per subject. The time points include sampling time points and dosing time points. The sample type includes blood, plasma, and urine. The drug quantification methods include HPLC/UV, LC/MS/MS, LC/MS, and radiographic.

CYP450 family enzymes predominantly exist in the gut wall and liver. Transporters are tissue specific. Table 5 presents the tissue specific transports and their functions. Probe drug is another important concept in the pharmacology research. An enzyme’s probe substrate means that this substrate is primarily metabolized or transported by this enzyme. In order to experimentally prove whether a new drug inhibits or induces an enzyme, its probe substrate is always utilized to demonstrate this enzyme’s activity before and after inhibition or induction. An enzyme’s probe inhibitor or inducer means that it inhibits or induces this enzyme primarily. Similarly, an enzyme’s probe inhibitor needs to be utilized if we investigate whether a drug is metabolized by this enzyme. Table 6 presents all the probe inhibitors, inducers, and substrates of CYP enzymes. Table 7 presents all the probe inhibitors, inducers, and substrates of the transporters. All these information were collected from industry standard (http://www.fda.gov/Drugs/GuidanceComplianceRegulatoryInformation/Guidances/ucm064982.htm), reviewed in the top pharmacology journal [9]. 

**Table 5:** Tissue specific transporters

**Gene**	**Aliases**	**Tissue type**	**Function**
*ABCB1*	P-gp, MDR1	Intestinal enterocyte, kidney proximal tubule, hepatocyte (canalicular), brain endothelia	Efflux
*ABCG2*	BCRP	Intestinal enterocyte, hepatocyte (canalicular), kidney proximal tubule, brain endothelia, placenta, stem cells, mammary gland (lactating)	Efflux
*SLCO1B1*	OATP1B1, OATP-C, OATP2, LST-1	Hepatocyte (sinusoidal)	Uptake
*SLCO1B3*	OATP1B3, OATP-8	Hepatocyte (sinusoidal)	Uptake
*SLC22A2*	OCT2	Kidney proximal tubule	Uptake
*SLC22A6*	OAT1	Kidney proximal tubule, placenta	Uptake
*SLC22A8*	OAT3	Kidney proximal tubule, choroid plexus, brain endothelia	Uptake

**Table 6:** ***In vivo *probe inhibitors/inducers/substrates of CYP enzymes**

**CYP enzymes**	**Inhibitors**	**Inducers**	**Substrates**
CYP1A2	Ciprofloxacin, enoxacin, fluvoxamine, Methoxsalen, mexiletine, oral contraceptives, phenylpropanolamine, thiabendazole, vemurafenib, zileuton, acyclovir, allopurinol, caffeine, cimetidine, daidzein, disulfiram, Echinacea, famotidine, norfloxacin, propafenone, propranolol, terbinafine, ticlopidine, verapamil	Montelukast, phenytoin, smokers versus non-smokers, moricizine, omeprazole, phenobarbital	Alosetron, caffeine, duloxetine, melatonin, ramelteon, tacrine, tizanidine, theophylline, tizanidine
CYP2B6	Clopidogrel, ticlopidine prasugrel	Efavirenz, rifampin, nevirapine	Bupropion, efavirenz
CYP2C8	Gemfibrozil, fluvoxamine, ketoconazole, trimethoprim	Rifampin	Repaglinide, Paclitaxel
CYP2C9	Amiodarone, fluconazole, miconazole, oxandrolone, capecitabine, cotrimoxazole, etravirine, fluvastatin, fluvoxamine, metronidazole, sulfinpyrazone, tigecycline, voriconazole, zafirlukast	Carbamazepine, rifampin, aprepitant, bosentan, phenobarbital, St. John’s wort	Celecoxib, Warfarin, phenytoin
CYP2C19	Fluconazole, fluvoxamine, ticlopidine, esomeprazole, fluoxetine, moclobemide, omeprazole, voriconazole, allicin (garlic derivative), armodafinil, carbamazepine, cimetidine, etravirine, human growth hormone (rhGH), felbamate, ketoconazole, oral contraceptives	Rifampin, artemisinin	Clobazam, lansoprazole, omeprazole, Smephenytoin, S-mephenytoin
CYP3A	Boceprevir, clarithromycin, conivaptan, grapefruit juice, indinavir, itraconazole,	Avasimibe, carbamazepine, phenytoin, rifampin, St. John’s wort, bosentan, efavirenz, etravirine, modafinil, nafcillin, amprenavir, aprepitant, armodafinil, clobazamechinacea, pioglitazone, prednisone, rufinamide, vemurafenib	Alfentanil, aprepitant, budesonide, buspirone, conivaptan, darifenacin, darunavir, dasatinib, dronedarone, eletriptan, eplerenone, everolimus, felodipine, indinavir, fluticasone, lopinavir, lovastatin, lurasidone, maraviroc, midazolam, nisoldipine, quetiapine, saquinavir, sildenafil, simvastatin, sirolimus, tolvaptan, tipranavir, triazolam, ticagrelor, vardenafil, Alfentanil, astemizole, cisapride, cyclosporine, dihydroergotamine, ergotamine, fentanyl, pimozide, quinidine, sirolimus, tacrolimus, terfenadine
	ketoconazole, lopinavir/ritonavir, mibefradil, nefazodone, nelfinavir, posaconazole, ritonavir, saquinavir, telaprevir, telithromycin, voriconazole, amprenavir, aprepitant, atazanavir, ciprofloxacin, crizotinib, darunavir/ritonavir, diltiazem, erythromycin, fluconazole, fosamprenavir, grapefruit juice, imatinib, verapamil, alprazolam, amiodarone, amlodipine, atorvastatin, bicalutamide, cilostazol, cimetidine, cyclosporine, fluoxetine, fluvoxamine, ginkgo, goldenseal, isoniazid, lapatinib, nilotinib, oral contraceptives, pazopanib, ranitidine, ranolazine, tipranavir/ritonavir, ticagrelor, zileuton		
CYP2D6	Bupropion, fluoxetine, paroxetine, quinidine, cinacalcet, duloxetine, terbinafine,	NA	Atomoxetine, desipramine, dextromethorphan, metoprolol, nebivolol, perphenazine, tolterodine, venlafaxine, Thioridazine, pimozide
	amiodarone, celecoxib, clobazam, cimetidine, desvenlafaxine, diltiazem, diphenhydramine, echinacea, escitalopram, febuxostat, gefitinib, hydralazine, hydroxychloroquine, imatinib, methadone, oral contraceptives, pazopanib, propafenone, ranitidine, ritonavir, sertraline, telithromycin, verapamil, vemurafenib		

**Table 7:** ***In vivo *probe inhibitors/inducers/substrates of selected transporters**

**Transporter**	**Inhibitor**	**Inducer**	**Substrate**
P-gp	Amiodarone, azithromycin, captopril, carvedilol, clarithromycin, conivaptan, cyclosporine, diltiazem, dronedarone, erythromycin, felodipine, itraconazole, ketoconazole, lopinavir and ritonavir, quercetin, quinidine, ranolazine, ticagrelor, verapamil	Avasimibe, carbamazepine, phenytoin, rifampin, St John’s wort, tipranavir/ritonavir	Aliskiren, ambrisentan, colchicine, dabigatran etexilate, digoxin, everolimus, fexofenadine, imatinib, lapatinib, maraviroc, nilotinib, posaconazole, ranolazine, saxagliptin, sirolimus, sitagliptin, talinolol, tolvaptan, topotecan
BCRP	Cyclosporine, elacridar (GF120918), eltrombopag, gefitinib	NA	Methotrexate, mitoxantrone, imatinib, irrinotecan, lapatinib, rosuvastatin, sulfasalazine, topotecan
OATP1B1	Atazanavir, cyclosporine, eltrombopag, gemfibrozil, lopinavir, rifampin, ritonavir, saquinavir, tipranavir	NA	Atrasentan, atorvastatin, bosentan, ezetimibe, fluvastatin, glyburide, SN-38 (active metabolite of irinotecan), rosuvastatin, simvastatin acid, pitavastatin, pravastatin, repaglinide, rifampin, valsartan, olmesartan
OATP1B3	Atazanavir, cyclosporine, lopinavir, rifampin, ritonavir, saquinavir	NA	Atorvastatin, rosuvastatin, pitavastatin, telmisartan, valsartan, olmesartan
OCT2	Cimetidine, quinidine	NA	Amantadine, amiloride, cimetidine, dopamine, famotidine, memantine, metformin, pindolol, procainamide, ranitidine, varenicline, oxaliplatin
OAT1	Probenecid	NA	Adefovir, captopril, furosemide, lamivudine, methotrexate, oseltamivir, tenofovir, zalcitabine, zidovudine
OAT3	Probenecid cimetidine, diclofenac	NA	Acyclovir, bumetanide, ciprofloxacin, famotidine, furosemide, methotrexate, zidovudine, oseltamivir acid, (the active metabolite of oseltamivir), penicillin G, pravastatin, rosuvastatin, sitagliptin

*Metabolism* The cytochrome P450 superfamily (officially abbreviated as CYP) is a large and diverse group of enzymes that catalyze the oxidation of organic substances. The substrates of CYP enzymes include metabolic intermediates such as lipids and steroidal hormones, as well as xenobiotic substances such as drugs and other toxic chemicals. CYPs are the major enzymes involved in drug metabolism and bioactivation, accounting for about 75% of the total number of different metabolic reactions [10]. CYP enzyme names and genetic variants were mapped from the Human Cytochrome P450 (CYP) Allele Nomenclature Database (http://www.cypalleles.ki.se/). This site contains the CYP450 genetic mutation effect on the protein sequence and enzyme activity with associated references.

*Transport Proteins* are proteins which serves the function of moving other materials within an organism. Transport proteins are vital to the growth and life of all living things. Transport proteins involved in the movement of ions, small molecules, or macromolecules, such as another protein, across a biological membrane. They are integral membrane proteins; that is they exist within and span the membrane across which they transport substances. Their names and genetic variants were mapped from the Transporter Classification Database (http://www.tcdb.org). In addition, we also added the probe substrates and probe inhibitors to each one of the metabolism and transportation enzymes (see prescribed description).

*Drug* names was created using the drug names from DrugBank 3.0 [11]. DrugBank consists of 6,829 drugs which can be grouped into different categories of FDA-approved, FDA approved biotech, nutraceuticals, and experimental drugs. The drug names are mapped to generic names, brand names, and synonyms.

*Subject* included the existing ontologies for human disease ontology (DOID), suggested Ontology for Pharmacogenomics (SOPHARM),, and mammalian phenotype (MP) from http://bioportal.bioontology.org (see Table 1)The PK ontology was implemented with Protégé [12] and uploaded to the BioPortal ontology platform.

### PK corpus

A PK abstract corpus was constructed to cover four primary classes of PK studies: clinical PK studies (n = 56); clinical pharmacogenetic studies (n = 57); *in vivo* DDI studies (n = 218); and *in vitro* drug interaction studies (n = 210). The PK corpus construction process is a manual process. The abstracts of clinical PK studies were selected from our previous work, in which the most popular CYP3A substrate, midazolam was investigated [4]. The clinical pharmacogenetic abstracts were selected based on the most polymorphic CYP enzyme, CYP2D6. We think these two selection strategies represent very well all the *in vivo* PK and PG studies. In searching for the drug interaction studies, the abstracts were randomly selected from a PubMed query, which used probe substrates/inhibitors/inducers for metabolism enzymes reported in the Table 6.

**Figure 1:**
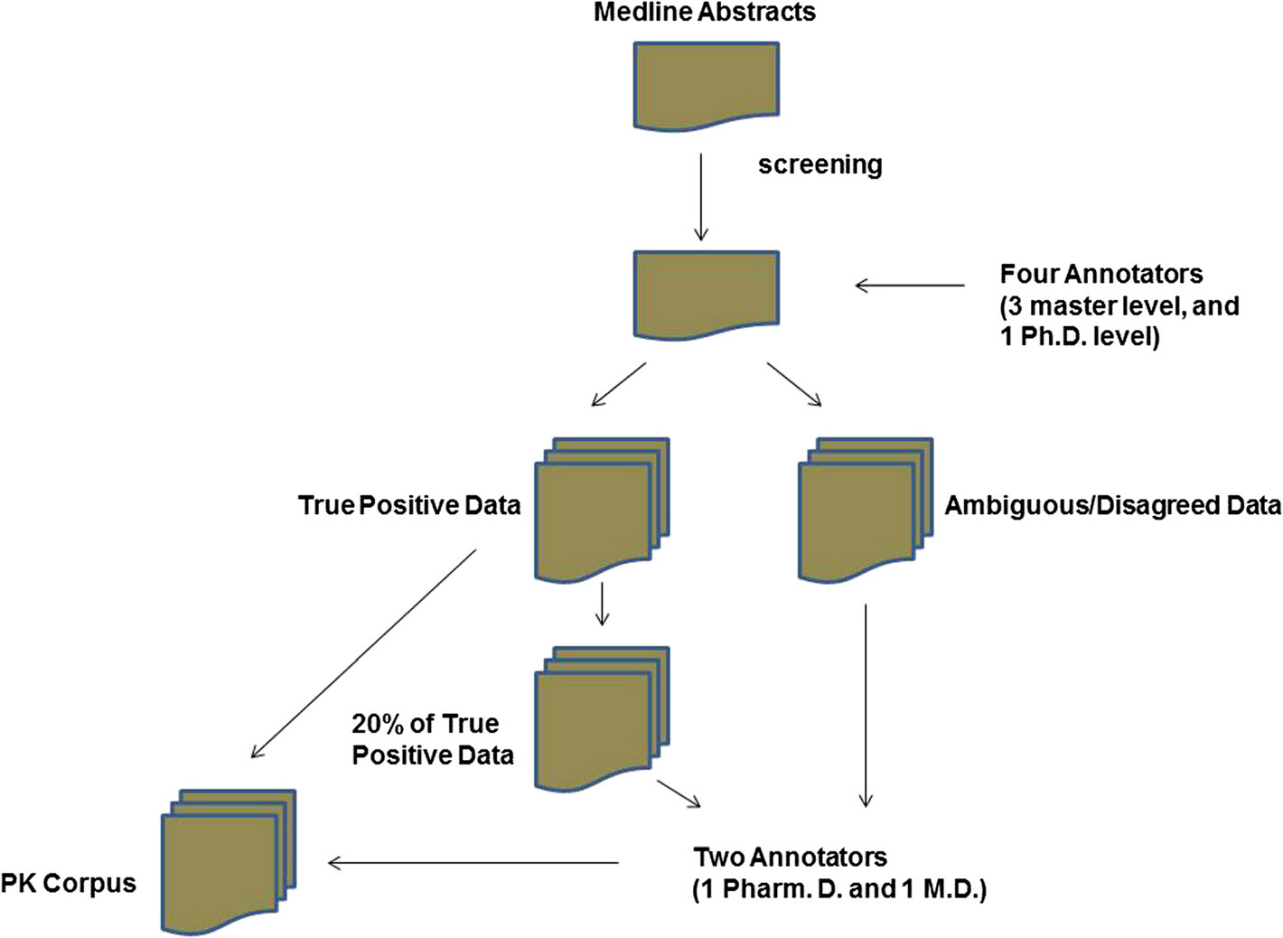
PK corpus annotation flow chart.

Once the abstracts have been identified in four classes, their annotation is a manual process (Figure 1). The annotation was firstly carried out by three master level annotators (Shreyas Karnik, Abhinita Subhadarshini, and Xu Han), and one Ph.D. annotator (Lang Li). They have different training backgrounds: computational science, biological science, and pharmacology. Any differentially annotated terms were further checked by Sara K. Quinney and David A. Flockhart, one Pharm D. and one M.D. scientists with extensive pharmacology training background. Among the disagreed annotations between these two annotators, a group review was conducted (Drs Quinney, Flockhart, and Li) to reach the final agreed annotations. In addition a random subset of 20% of the abstracts that had consistent annotations among four annotators (3 masters and one Ph.D.), were double checked by two Ph.D. level scientists.

**Figure 2:**
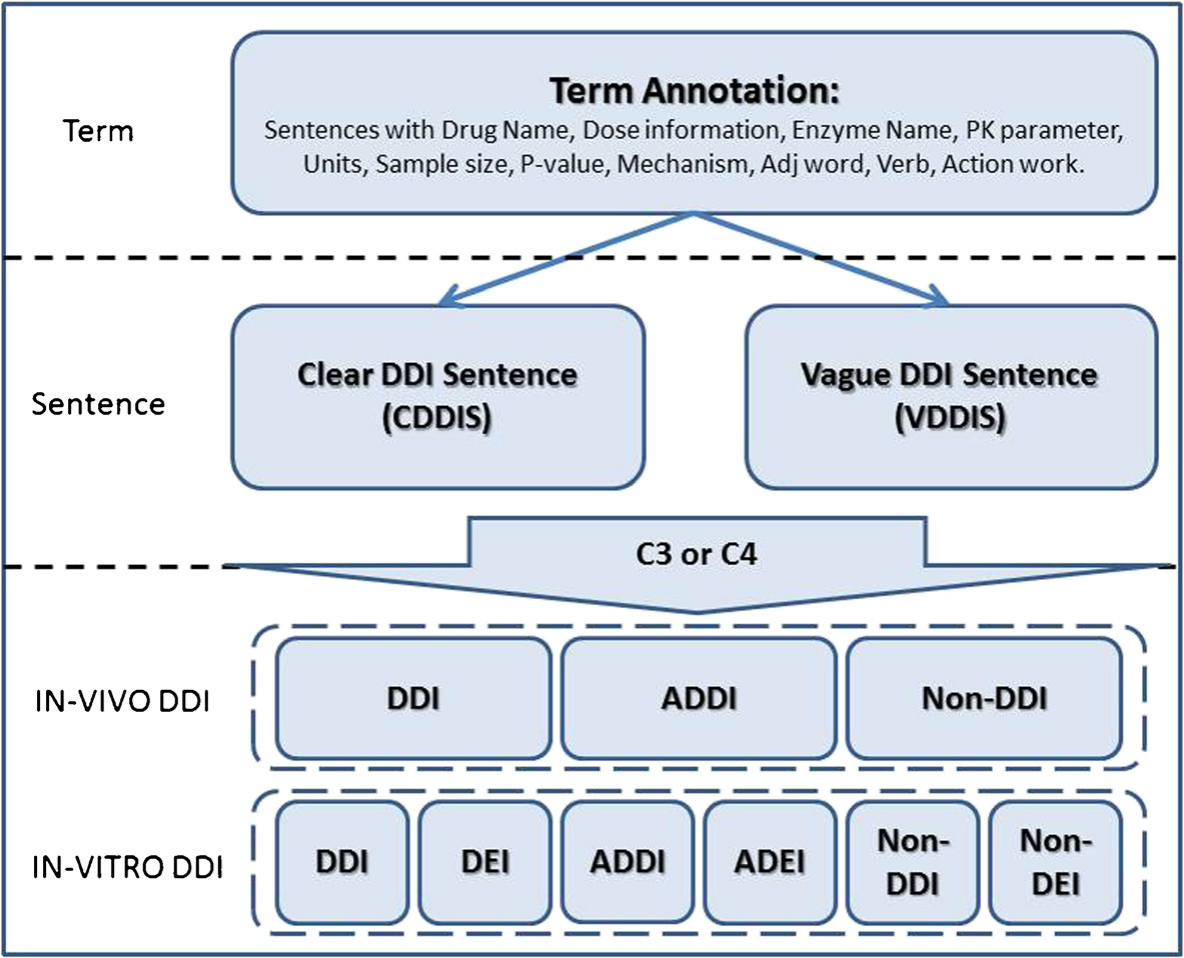
A three level hierarchical PK and DDI annotation scheme.

A structured annotation scheme was implemented to annotate three layers of pharmacokinetics information: key terms, DDI sentences, and DDI pairs (Figure 2). DDI sentence annotation scheme depends on the key terms; and DDI annotations depend on the key terms and DDI sentences. Their annotation schemes are described as following.

Key terms include drug names, enzyme names, PK parameters, numbers, mechanisms, and change. The boundaries of these terms among different annotators were judged by the following standard.

• *Drug names* were defined mainly on DrugBank 3.0 [11]. In addition, drug metabolites were also tagged, because they are important in *in vitro* studies. The metabolites were judged by either prefix or suffix: oxi, hydroxyl, methyl, acetyl, N-dealkyl, N-demethyl, nor, dihydroxy, O-dealkyl, and sulfo. These prefixes and suffixes are due to the reactions due to phase I metabolism (oxidation, reduction, hydrolysis), and phase II metabolism (methylation, sulphation, acetylation, glucuronidation) [13].

• *Enzyme names* covered all the CYP450 enzymes. Their names are defined in the human cytochrome P450 allele nomenclature database, http://www.cypalleles.ki.se/. The variations of the enzyme or gene names were considered. Its regular expression is (?:cyp|CYP|P450|CYP450)?[0–9][a-zA-Z][0–9](?:\*[0–9])?$.

• *PK parameters* were annotated based on the defined *in vitro* and *in vivo* PK parameter ontology in Table 2 and 4. In addition, some PK parameters have different names, CL = clearance, t1/2 = half-life, AUC = area under the concentration curve, and AUCR = area under the concentration curve ratio.

• *Numbers* such as dose, sample size, the values of PK parameters, and p-values were all annotated. If presented, their units were also covered in the annotations.

• *Mechanisms* denote the drug metabolism and interaction mechanisms. They were annotated by the following regular expression patterns: inhibit(e(s|d)?|ing|ion(s)?|or)$, catalyz(e(s|d)?|ing)$, correlat(e(s|d)?|ing|ion(s)?)$, metaboli(z(e(s|d)?|ing)|sm)$, induc(e(s|d)?|ing|tion(s)?|or)$, form((s|ed)?|ing|tion(s)?|or)$, stimulat(e(s|d)?|ing|ion(s)?)$, activ(e(s)?|(at)(e(s|d)?|ing|ion(s)?))$, and suppress(e(s|d)?|ing|ion(s)?)$.

• *Change* describes the change of PK parameters. The following words were annotated in the corpus to denote the change: strong(ly)?, moderate(ly)?, high(est)?(er)?, slight(ly)?, strong(ly)?, moderate(ly)?, slight(ly)?, significant(ly)?, obvious(ly)?, marked(ly)?, great(ly)?, pronounced(ly)?, modest(ly)?, probably, may, might, minor, little, negligible, doesn’t interact, affect((s|ed)?|ing|ion(s)?)?$, reduc(e(s|d)?|ing|tion(s)?)$, and increas(e(s|d)?|ing)$.

The middle level annotation focused on the drug interaction sentences. Because two interaction drugs were not necessary all presented in the sentence, sentences were categorized into two classes:

• Clear DDI Sentence (CDDIS): two drug names (or drug-enzyme pair in the *in vitro* study) are in the sentence with a clear interaction statement, i.e. either interaction, or non-interaction, or ambiguous statement (i.e. such as possible or might and etc.).

• Vague DDI Sentence (VDDIS): One drug or enzyme name is missed in the DDI sentence, but it can be inferred from the context. Clear interaction statement also is required.

Once DDI sentences were labeled, the DDI pairs in the sentences were further annotated. Because the fundamental difference between *in vivo* DDI studies and *in vitro* DDI studies, their DDI relationships were defined differently. In *in vivo* studies, three types of DDI relationships were defined (Table 8): DDI, ambiguous DDI (ADDI), and non-DDI (NDDI). Four conditions are specified to determine these DDI relationships. Condition 1 (C1) requires that at least one drug or enzyme name has to be contained in the sentence; condition 2 (C2) requires the other interaction drug or enzyme name can be found from the context if it is not from the same sentence; condition 3 (C3) specifies numeric rules to defined the DDI relationships based on the PK parameter changes; and condition 4 (C4) specifies the language expression patterns for DDI relationships. Using the rules summarized in Table 8, DDI, ADDI, and NDDI can be defined by C1 ^ C2 ^ (C3 ^ C4). The priority rank of *in vivo* PK parameters is AUC > CL > t_1/2_ > C_max_. In *in vitro* studies, six types of DDI relationships were defined (Table 8). DDI, ADDI, NDDI were similar to *in vivo* DDIs, but three more drug-enzyme relationships were further defined: DEI, ambiguous DEI (ADEI), and non-DDI (NDEI). C1, C2, and C4 remained the same for *in vitro* DDIs. The main difference is in C3, in which either Ki or IC50 (inhibition) or EC50 (induction) were used to defined DDI relationship quantitatively. The priority rank of *in vitro* PK parameters is Ki > IC50. Table 9 presented eight examples of how DDIs or DEIs were determined in the sentences.

**Table 8:** DDI definitions in corpus

**DDI relationship**	**C1**	**C2**	C3**	C4**
**IN VIVO STUDY**				
**DDI**	**Yes**	**Yes**	The PK parameter with the highest priority* must satisfy p-value <0.05 and FC > 1.50 or FC < 0.67	Significant, obviously, markedly, greatly, pronouncedly and etc.
**Ambiguous DDI (ADDI)**			The PK parameter with the highest priority* in the conditions of p-value <0.05 but 0.67 < FC < 1.50; or FC >1.50 or FC <0.67, but p-value > 0.05.	Modestly, moderately, probably, may, might, and etc.
**Non-DDI (NDDI)**			The PK parameter with the highest priority*are in the condition of p-value > 0.05 and 0.67 < FC < 1.50	Minor significance, slightly, little or negligible effect, doesn’t interact etc.
**IN VITRO STUDY**				
**DDI**	**Yes**	**Yes**	(0< Ki < 10 or 0< EC50 < 10 microM, and p-value <0.05)	Significant, obviously, markedly, greatly, pronouncedly and etc.
**DEI**				
**Ambiguous DDI (ADDI)**			(10 < Ki < 100 or 10 < EC50 < 100 microM, and p-value <0.05 or vice versa)	Modestly, moderately, probably, may, might, and etc.
**Ambiguous DEI (ADEI)**				
**Non-DDI (NDDI)**			(Ki > 100 microM or EC50 > 100 microM, and p-value >0.05)	Minor significance, slightly, little or negligible effect, doesn’t interact etc.
**Non-DEI (NDEI)**				

Note:

C1: At least one drug or enzyme name has to be contained in the sentence.

C2: Need to label the drug name if it is not from the same sentence.

C3: PK-parameter and value dependent.

C4: Significance statement.

*Priority issue: When C3 and C4 occur and conflict, C3 dominates the sentence.**For the priority of PK parameters: AUC > CL > t_1/2_ > C_max;_; the priority of *in vitro* PK parameters: Ki>IC50.

**Table 9:** Examples of DDI definitions

**PMID**	**DDI sentence**	**Relationship and commend**
**20012601**	The pharmacokinetic parameters of *verapamil* were *significantly* altered by the co-administration of *lovastatin* compared to the control.	Because of the words, “significantly”, (Verapamil, lovastatin) is a **DDI**.
**20209646**	The *clearance* of *mitoxantrone* and *etoposide* was *decreased* by *64%* and *60%*, respectively, when combined with *valspodar*.	Because of the fold changes were less than 0.67, (*mitoxantrone, valspodar*.) and (*etoposide, valspodar*) are **DDIs**.
**20012601**	The *(AUC (0-infinity))* of *norverapamil* and the terminal *half-life* of *verapamil did not significantly changed* with *lovastatin* coadministration.	Because of the words, “not significantly changed”, *(verapamil*, *ovastatin*) is a **NDDI**.
**17304149**	Compared with placebo, *itraconazole* treatment *significantly increase* the peak plasma concentration (*Cmax*) of paroxetine by 1.3 fold (6.7 2.5 versus 9.0 3.3 ng/mL, P≤0.05) and the area under the plasma concentration-time curve from zero to 48 hours [*AUC(*0*–*48)] of *paroxetine* by *1.5 fold* (137 73 versus 199 91 ng*h/mL, *P≤0.01*).	AUC has a higher rank than Cmax, and it had a 1.5 fold-change and less than 0.05 p-value, thus, (*itraconazole*, *paroxetine)* is a **DDI**.
**13129991**	The mean (SD) *urinary ratio* of *dextromethorphan* to its metabolite was *0.006* (0.010) at baseline and *0.014* (0.025) after *St John’s wort* administration *(P=.26)*	The change in PK parameter is more than 1.5 fold but P-value is >0.05. Thus, (dextromethorphan, St John’s wort) is an **ADDI**.
**19904008**	The obtained results show that *perazine* at its therapeutic concentrations is a *potent inhibitor* of human *CYP1A2.*	Because of words, “potent inhibitor”, (perazine, CYP1A2) is a **DEI**.
**19230594**	After human hepatocytes were exposed to 10 microM *YM758*, microsomal activity and mRNA level for *CYP1A2* were *not induced* while those for *CYP3A4* were *slightly induced*.	Because of words, “not induced” and “slightly induced”, (YM758, CYP1A2) and (YM758, CYP1A2) are **NDEIs**.
**19960413**	From these results, *DPT* was characterized to be a competitive *inhibitor* of *CYP2C9* and *CYP3A4*, with *K(i)* values of *3.5* and *10.8 microM* in HLM and *24.9* and *3.5* microM in baculovirus-insect cell-expressed human CYPs, respectively.	Because K was larger than 10microM, (DPT, CYP2C9) and (DPT, CYP3A4) are **ADEIs**.

Krippendorff’s alpha [14] was calculated to evaluate the reliability of annotations from four annotators. The frequencies of key terms, DDI sentences, and DDI pairs are presented in Table 10. Their Krippendorff’s alphas are 0.953, 0.921, and 0.905, respectively. Please note that the total DDI pairs refer to the total pairs of drugs within a DDI sentence from all DDI sentences.

**Table 10:** Annotation performance evaluation

**Key Terms**	**Annotation Categories**	**Frequencies**	**Krippendorff’s alpha**
	Drug	8633	0.953
	CYP	3801	
	PK Parameter	1508	
	Number	3042	
	Mechanism	2732	
	Change	1828	
	Total words	97291	
DDI sentences	CDDI sentences	1191	0.921
	VDDI sentences	120	
	Total sentences	4724	
DDI Pairs	DDI	1239	0.905
	ADDI	300	
	NDDI	294	
	DEI	565	
	ADEI	95	
	NDEI	181	
	Total Drug Pairs	12399	

The PK corpus was constructed by the following process. Raw abstracts were downloaded from PubMed in XML format. Then XML files were converted into GENIA corpus format following the gpml.dtd from the GENIA corpus [15]. The sentence detection in this step is accomplished by using the Perl module Lingua::EN::Sentence, which was downloaded from The Comprehensive Perl Archive Network (CPAN, http://www.cpan.org). GENIA corpus files were then tagged with the prescribed three levels of PK and DDI annotations. Finally, a cascading style sheet (CSS) was implemented to differentiate colours for the entities in the corpus. This feature allows the users to visualize annotated entities. We would like to acknowledge that a DDI Corpus was recently published as part of a text mining competition DDIExtraction 2011 (http://labda.inf.uc3m.es/ DDIExtraction2011/dataset.html). Their DDIs were clinical outcome oriented, not PK oriented. They were extracted from DrugBank, not from PubMed abstracts. Our PK corpus complements to their corpus very well.

## Utility

### Example 1: An annotated tamoxifen pharmacogenetics study

This example shows how to annotate a pharmacogenetics studies with the PK ontology. We used a published tamoxifen PG study [16]. The key information from this tamoxifen PG trial was extracted as a summary list. Then the pre-processed information was mapped to the PK ontology (column 2 in Additional file 1: Table S1). This PG study investigates the genetics effects (CYP3A4, CPY3A5, CYP2D6, CYP2C9, CYP2B6) on the tamoxifen pharmacokinetics outcome (tamoxifen metabolites) among breast cancer patients. It was a single arm longitudinal study (n = 298), patients took SOLTAMOX^TM^ 20mg/day, and the drug steady state concentration was sampled (1, 4, 8, 12) months after the tamoxifen treatment. The study population was a mixed Caucasian and African American. In additional file 1: Table S1, the trial summary is well organized by the PK ontology.

### Example 2 midazolam/ketoconazole drug interaction study

This was a cross-over three-phase drug interaction study [17] (n = 24) between midazolam (MDZ) and ketoconazole (KTZ). Phase I was MDZ alone (IV 0.05 mg/kg and PO 4mg); phase II was MDZ plus KTZ (200mg); and phase III was MDZ plus KTZ (400mg). Genetic variable include CYP3A4 and CYP3A5. The PK outcome is the MDZ AUC ratio before and after KTZ inhibition. Its PK ontology based annotation is shown in Additional file 1: Table S1 column three.

### Example 3 *in vitro* Pharmacokinetics Study

This was an *in vitro* study [18], which investigated the drug metabolism activities for 3 enzymes, such as CYP3A4, CYP3A5, and CYP3A7 in a recombinant system. Using 10 CYP3A substrates, they compared the relative contribution of 3 enzymes among 10 drug’s metabolism. Its PK ontology based annotation is shown in Additional file 1: Table S2.

### Example 4 A drug interaction text mining example

We implemented the approach described by [19] for the DDI extraction. Prior to performing DDI extraction, the testing and validation DDI abstracts in our corpus was pre-processed and converted into the unified XML format [19]. The following steps were conducted:

• Drugs were tagged in each of the sentences using dictionary based on DrugBank. This step revised our prescribed drug name annotations in the corpus. One purpose is to reduce the redundant synonymous drug names. The other purpose is only keep the parent drugs and remove the drug metabolites from the tagged drug names from our initial corpus, because parent drugs and their metabolites rarely interacts. In addition, enzymes (i.e. CYPs) were also tagged as drugs, since enzyme-drug interactions have been extensively studied and published. The regular expression of enzyme names in our corpus was used to remove the redundant synonymous gene names.

• Each of the sentences was subjected to tokenization, PoS tags and dependency tree generation using the Stanford parser [20].

• C_2_^*n *^drug pairs form the tagged drugs in a sentence were generated automatically, and they were assigned with default labels as no-drug interaction. Please note that if a sentence had only one drug name, this sentence didn’t have a DDI. This setup limited us considering only CDDI sentence in our corpus.

• The drug interaction labels were then manually flipped based on their true drug interaction annotations from the corpus. Please note that our corpus had annotated DDIs, ADDIs, NDDIs, DEIs, ADEIs, and NDEIs. Here only DDIs and DEIs were labeled as true DDIs. The other ADDIs, NDDIs, DEIs, and ADEIs were all categorized into the no-drug interactions.

**Figure 3:**
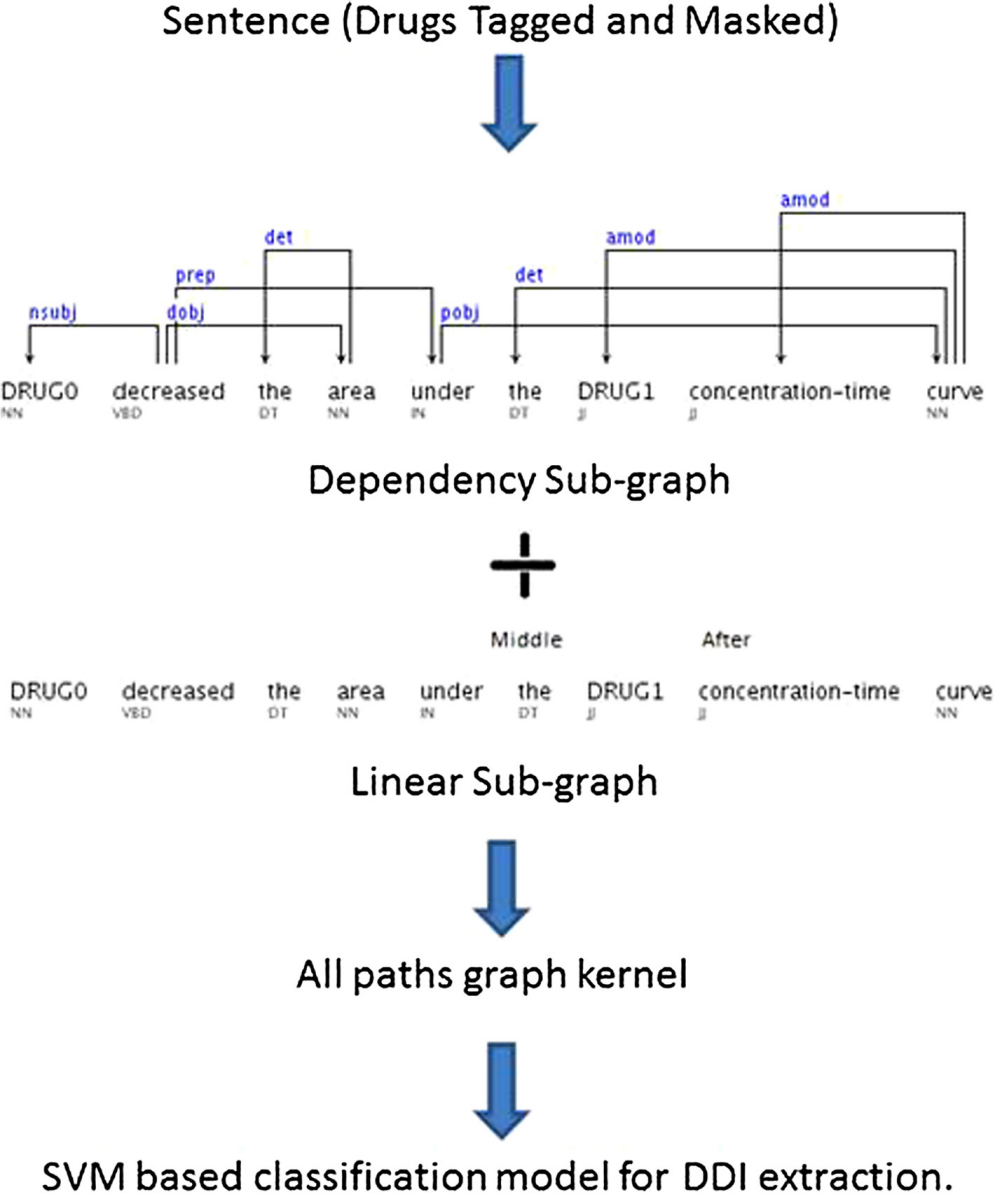
Drug interaction extraction algorithm flow chart.

Then sentences were represented with dependency graphs using interacting components (drugs) (Figure 3). The graph representation of the sentence was composed of two items: i) One dependency graph structure of the sentence; ii) a sequence of PoS tags (which was transformed to a linear order “graph” by connecting the tags with a constant edge weight). We used the Stanford parser [20] to generate the dependency graphs. Airola et al. proposed to combine these two graphs to one weighted, directed graph. This graph was fed into a support vector machine (SVM) for DDI/non-DDI classification. More details about the all paths graph kernel algorithm can be found in [19]. A graphical representation of the approach is presented in Figure 3.

DDI extraction was implemented in the *in vitro* and *in vivo* DDI corpus separately. Table 11 presented the training sample size and testing sample size in both corpus sets. Then Table 12 presents the DDI extraction performance. In extracting *in vivo* DDI pairs, the precision, recall, and F-measure in the testing set are 0.67, 0.79, and 0.73, respectively. In the *in vitro* DDI extraction analysis, the precision, recall, and F-measure are 0.47, 0.58, 0.52 respectively in the *in vitro* testing set. In our early DDI research published in the DDIExtract 2011 Challenge [21], we used the same algorithm to extract both *in vitro* and *in vivo* DDIs at the same time, the reported F-measure was 0.66. This number is in the middle of our current *in vivo* DDI extraction F-measure 0.73 and *in vitro* DDI extraction F-measure 0.52.

**Table 11:** DDI data description

**Datasets**	**Abstracts**	**Sentences**	**DDI pairs**	**True DDI pairs**
*in vivo* DDI training	174	2112	2024	359
*in vivo* DDI testing	44	545	574	45
*in vitro* DDI training	168	1894	7122	783
in vitro DDI testing	42	475	1542	146

**Table 12:** DDI extraction performance

**Datasets**	**precision**	**recall**	**f-measure**
*in vivo* DDI Training	0.67	0.78	0.72
*in vivo* DDI Testing	0.67	0.79	0.73
in vitro DDI Training	0.51	0.59	0.55
in vitro DDI Testing	0.47	0.58	0.52

Error analysis was performed in testing samples. Table 13 summarized the results. Among the known reasons for the false positives and false negatives, the most frequent one is that there are multiple drugs in the sentence, or the sentence is long. The other reasons include that there is no direct DDI relationship between two drugs, but the presence of some words, such as dose, increase, and etc., may lead to a false positive prediction; or DDI is presented in an indirect way; or some NDDI are inferred due to some adjectives (little, minor, negligible).

**Table 13:** DDI extraction error analysis from testing DDI sets

**No.**	**Error categories**	**Error type**	**Frequency**		**examples**
			**In vivo**	**In vitro**	
1	There are multiple drugs in the sentence, and the sentence is long.	FP	6	34	PMID: 12426514. In 3 subjects with measurable concentrations in the single-dose study, rifampin significantly decreased the mean maximum plasma concentration (C(max)) and area under the plasma concentration-time curve from 0 to 24 h [AUC(0–24)] of praziquantel by 81% (P <.05) and 85% (P <.01), respectively, whereas rifampin significantly decreased the mean C(max) and AUC(0–24) of praziquantel by 74% (P <.05) and 80% (P <.01), respectively, in 5 subjects with measurable concentrations in the multiple-dose study
		FN	2	17	PMID: 10608481. Erythromycin and ketoconazole showed a clear inhibitory effect on the 3-hydroxylation of lidocaine at 5 microM of lidocaine (IC50 9.9 microM and 13.9 microM, respectively), but did not show a consistent effect at 800 microM of lidocaine (IC50 >250 microM and 75.0 microM, respectively).
2	There is no direct DDI relationship between two drugs, but the presence of some words, such as dose, increase, and etc. may lead to a false positive prediction	FP	6	14	PMID: 17192504. A significant fraction of patients to be treated with HMR1766 is expected to be maintained on warfarin
3	DDI is presented in an indirect way.	FN	2	19	PMID: 11994058. In CYP2D6 poor metabolizers, systemic exposure was greater after chlorpheniramine alone than in extensive metabolizers, and administration of quinidine resulted in a slight increase in CLoral.
4	Design issue. Some NDDI are inferred due to some adjectives (little, minor, negligible)	FP	1	3	PMID: 10223772. In contrast,the effect of ranitidine or ebrotidine on CYP3A activity *in vivo* seems to have little clinical significance.
5	Unknown	FP	5	44	PMID: 10383922. CYP1A2, CYP2A6, and CYP2E1 activities were not significantly inhibited by azelastine and the two metabolites.
		FN	6	26	PMID: 10681383. However, the most unusual result was the interaction between testosterone and nifedipine.

## Conclusions and discussions

A comprehensive PK ontology was constructed. It annotates both *in vitro* PK experiments and *in vivo* PK studies. Using our PK ontology, a PK corpus was also developed. It consists of four classes of PK studies: *in vivo* PK studies, *in vivo* PG studies, *in vivo* DDI interaction studies, and *in vitro* DDI studies. This PK corpus is a highly valuable resource for text mining drug interactions relationship.

We previously had developed entity recognition algorithm or tools to tag PK parameters and their associated numerical data [4]. We had shown that for one drug, midazolam, we have achieved very high accuracy and recall rate in tagging PK parameter, clearance (CL), and its associated numerical values. However, using our newly developed PK corpus, we cannot regain such a good performance in a more general class of drugs and PK parameters. This area will need much further investigation.

We would like to acknowledge that a DDI Corpus was recently published as part of a text mining competition DDIExtraction 2011 (http://labda.inf.uc3m.es/DDIExtraction2011/dataset.html). Their DDIs were clinical outcome oriented, not PK oriented. They were extracted from DrugBank, not from PubMed abstracts. Our PK corpus complements to their corpus very well.

## Availability and requirements

PK ontology is available in OWL for download at http://rweb.compbio.iupui.edu/corpus/ontology/, which can be accessed by using any OWL editor/viewer, e.g., protégé. PK corpuses are available in XML at http://rweb.compbio.iupui.edu/corpus/.

## Abbreviation

ADMET: Absorption, disposition, metabolism, excretion, and transportation; DDI: Drug-drug interaction; KTZ: Ketoconazole; MDZ: Midazolam; POS: Part of speech; PK: Pharmacokinetics; PG: Pharmacogenetics.

## Competing interests

The authors declare that they have no competing interests.

## Authors’ contributions

H-YW developed the three level hierarchical PK and DDI annotation scheme for the corpus; SK designed the PK corpus annotation implementation scheme and was one of the master annotator; AS designed the PK ontology and was one of the master annotator; ZW applied the PK ontology to three PK studies; SP collected the pharmacogenetics abstracts; Xu Han was one of the master annotator; Chienwei Chiang collect the ontology information for the transporter; LLiu advised the utility of protégé; MB, LMR and SKQ defined the *in vitro* and *in vivo* PK terminologies; SKQ was one of the Ph.D. level annotator; DF confirmed the disagreed annotations and double checked the PK terminologies and study design; and LLi contributed the idea, guide this research, and wrote the manuscript. All authors read and approved the final manuscript.

## Supplementary Material

Additional file 1: Table S1 Clinical PK Studies. **Table S2.***in vitro* PK studies. Click here for file
