# Supplementary material for: An integrated pharmacokinetics ontology and corpus for text mining
Source: BMC Bioinformatics. 2013 Feb 1;14:35. doi: 10.1186/1471-2105-14-35 (PMC3571923; doi:10.1186/1471-2105-14-35)
Supplement: Additional file 1: Table S1 — Clinical PK Studies. Table S2.in vitro PK studies. [file 1471-2105-14-35-S1.pdf]

Table S1 Clinical PK Studies

Ontology	Pharmacogenetics Trial	Drug Interaction Trail
Drugs ≡ SOPHARM_20000	Tamoxifen (TAM)	Midazolam (MDZ, PO 4mg; IV 0.05mg/kg), Ketoconazole (KTZ, PO, 200, 400 mg)
Experiments		
in-vitro		
in-vivo	*in-vivo*	*in-vivo*
Analysis_Method		
Assay	HPLC/MS	HPLC/MS
Dose	SOLTAMOX™, 20mg/day	MDZ PO, IV; KTZ PO
Measurement	month 1, 4, 8, 12	before and 0.5, 0.75, 1, 2, 4, 6, 9 hrs
PK_Parameters	TAM and its metabolites conc	MDZ and KTZ: AUC, AUCR, $t_{1/2}$, and Cmax
Pre-dosing_Conditions		
Sample		
Sample_Size	298	24
Sample_Types	Blood	blood
Stratification	prior chemo, menopausal	
Study_Design		
Bioequivalence_Study		
Dense_Sampling		
Disease-Physiology_PK_Study		
Drug_Interaction_Study		inhibition
Longitudinal	Longitudinal	three-phase crossover
Pharmacogenetics_Study	prospective, single arm	prospective, single arm
Sparse_Sampling		
Steady_State_Study	steady state	
Type_of_PK_Study		
Metabolism		
CYP1_family		
CYP2_family	CYP2D6, 2C9, 2B6	
CYP3_family	CYP3A4/5	CYP3A4/5
CYP4_family		
CYP_other_families		
Subjects		
Disease ≡ DOID_14974	breast cancer	healthy volunteers
Physiology ≡ MP_0000001		
Population ≡ SOPHARM_52000	Caucasian/African American	
Target	ESR1/ESR2	

Note: The annotations are aligned for each row. The left column is the ontology tree presentation. The central and right columns display their corresponding annotations from the paper.

Table S2 *in vitro* PK Study

Ontology	in-vitro study
Drugs ≡ SOPHARM_20000	MDZ, APZ, TZ, CLAR, TAM, DTZ, NIF, BFC, HFC, TEST, E2
Experiments	
– in-vitro	Compare metabolic capabilities of CYP3A4, 3A5, 3A7
– – Experimental_Conditions	
– – – Buffer	
– – – NADPH_Source	sodium phosphate, NADPH, methanol.
– – – Other_Information	
– – – – Data_analysis_method	
– – – – Dilution	WinNonlin
– – – – Incubation_time	4 fold, 10% methanol (TZ)
– – – – Microsomal_binding	5 min
– – – – Number_of_replicates	insect cell (CYP3A)
– – – – Preincubation_time	N/A
– – – – Quantification_method	3min; 6 min
– – – – kdeg_or_ksyn_of_the_enzyme	HPLC, MS, Fluorimetry
– – – Protein	CYP3A4/5/7, P450 reductase, b5
– – – – Protein_Concentration	1mol, 6.6mol, 9mol
– – – – Source	BD Gentest, PanVera, PanVera
– – – – – Non_Recombinant-Enzymes	
– – – – – Recombinant_Enzymes	CYP3A
– – Inhibitor_or_Inducer	
– – Multi_Drug_Experiments	
– – – PK_Parameters	
– – – – Emax	
– – – – IC50	
– – – – KI	
– – – – Ki	
– – – – Kinact	
– – – Type_of_Interaction	
– – Single_Drug_Experiments	
– – – PK_Paramaters	
– – – – CLint	CL for individual substrates
– – – – Km	Km for individual substrates
– – – – Vmax	Vmax for individual substrates
– – Substrate	MDZ, APZ, TZ, CLAR, TAM, DTZ, NIF, BFC, HFC, TEST, E2
– in-vivo	
Metabolism	
– CYP1_family	
– CYP2_family	
– CYP3_family	CYP3A4, 3A5, 3A7
– CYP4_family	
– CYP_4_families_other	

Note: The annotations are aligned for each row. The left column is the ontology tree presentation. The central and right columns display their corresponding annotations from the paper.
